# Supplementary figures and images for: Patterns of fish utilisation in a tropical Indo-Pacific mangrove-coral seascape, New Caledonia
Source: PLoS One. 2019 Apr 19;14(4):e0207168. doi: 10.1371/journal.pone.0207168 (PMC6474647; doi:10.1371/journal.pone.0207168)

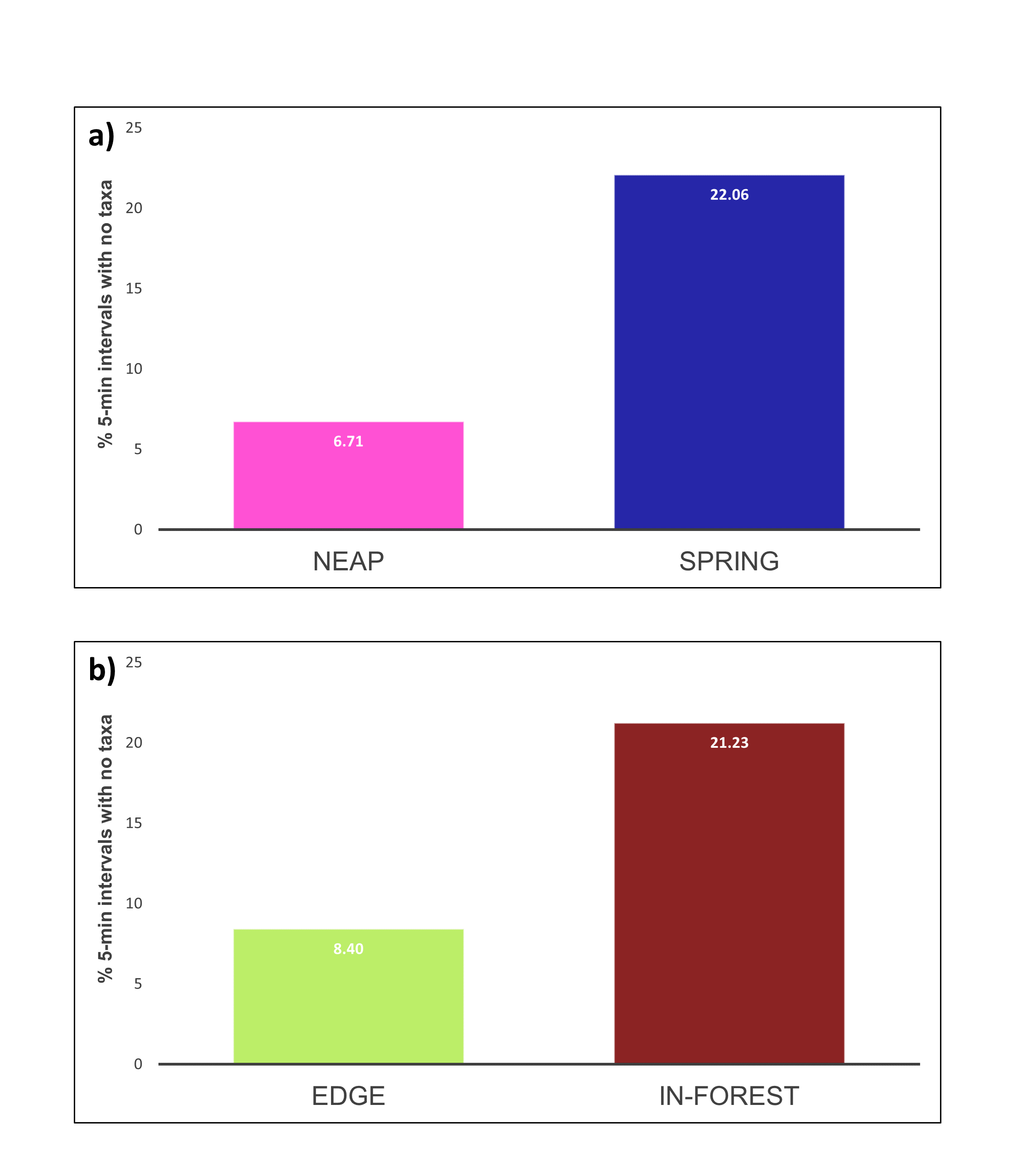

Supplement: S2 Appendix — Percentage of 5-minutes intervals with no common taxa observed at a) neap tide vs spring tide and b) edge vs in-forest habitats. (TIF) [file pone.0207168.s002.tif]

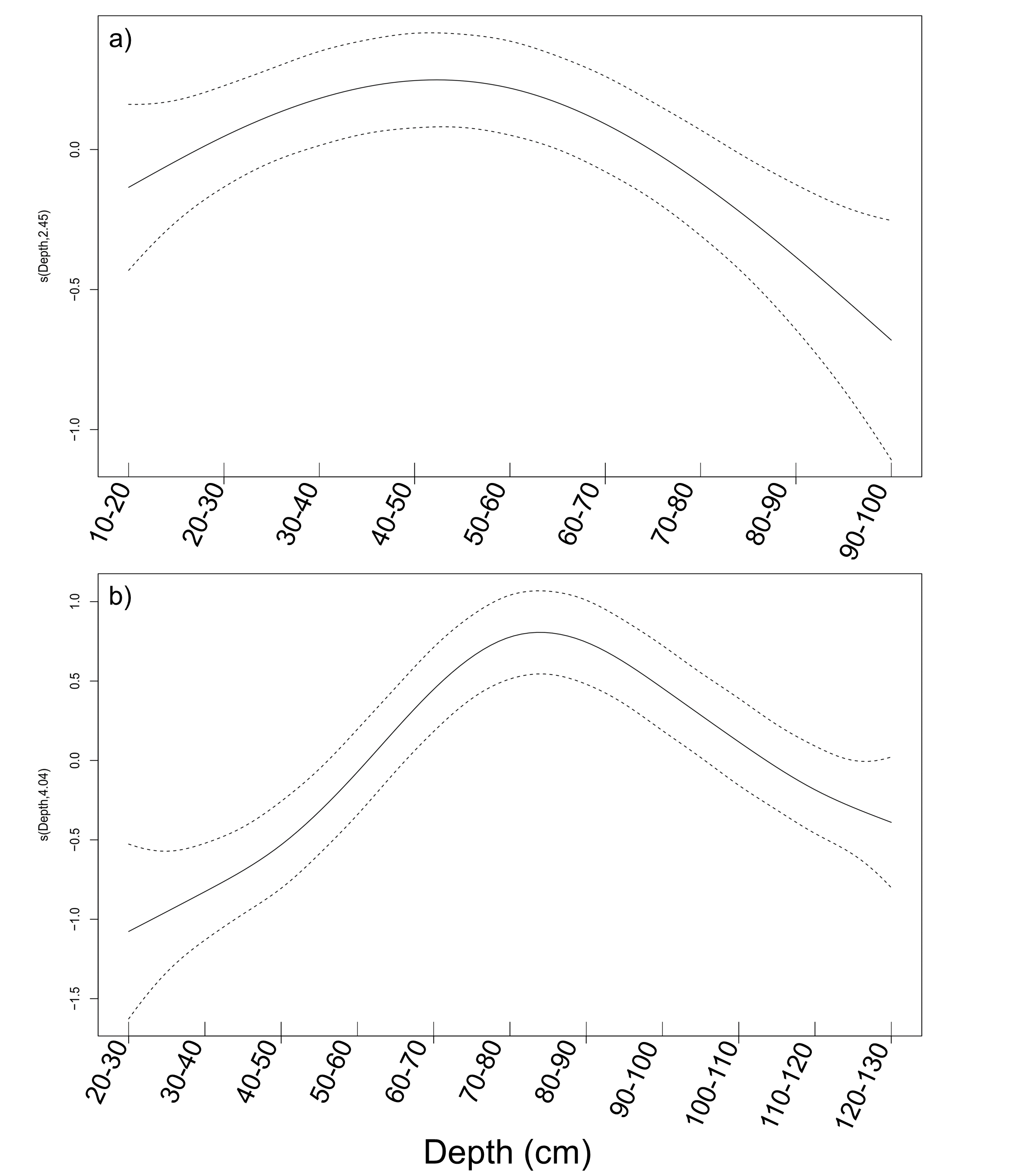

Supplement: S3 Appendix — General Additive Mixed Model for a) edge sites and b) in-forest sites. (TIF) [file pone.0207168.s003.tif]

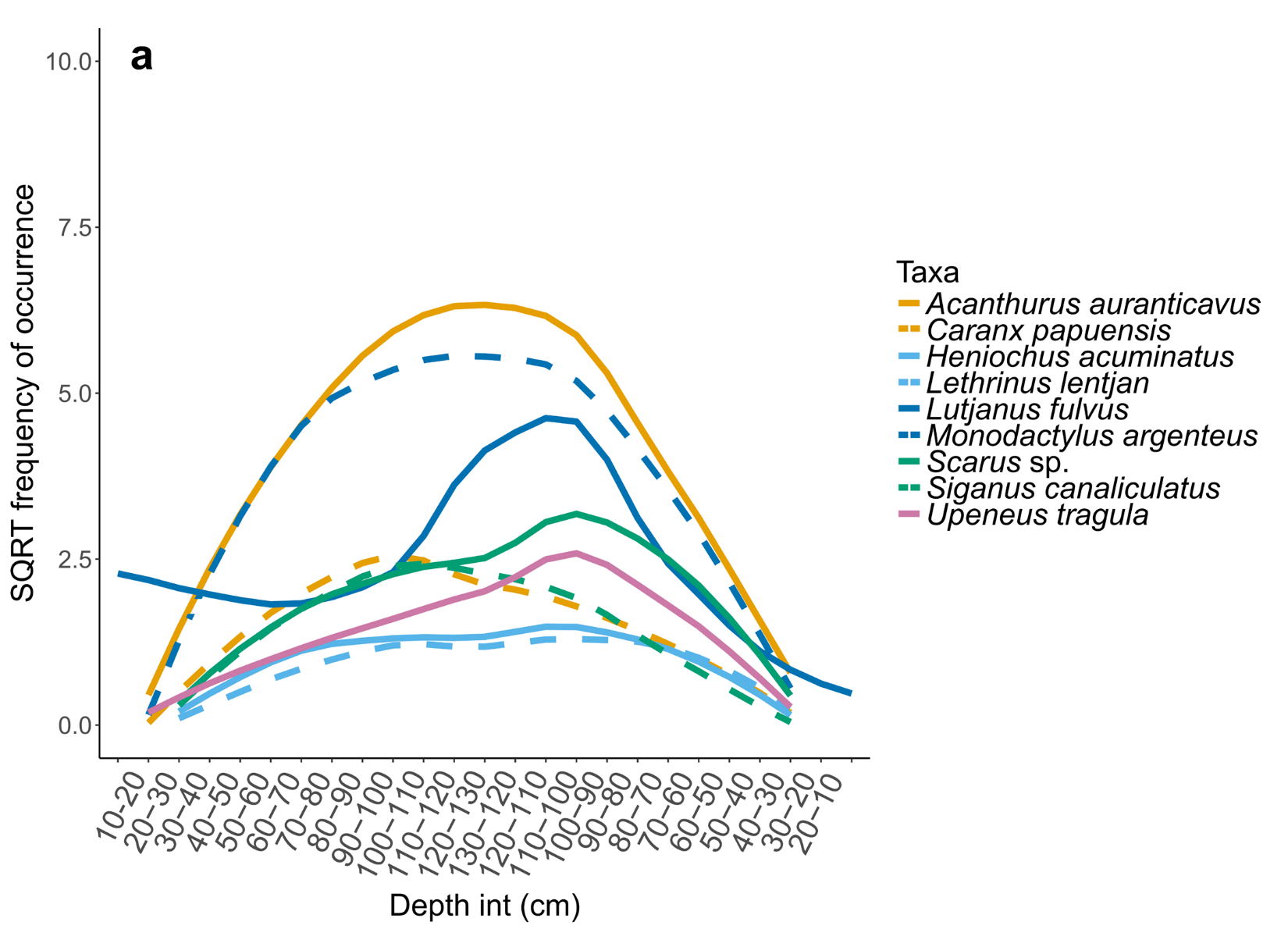

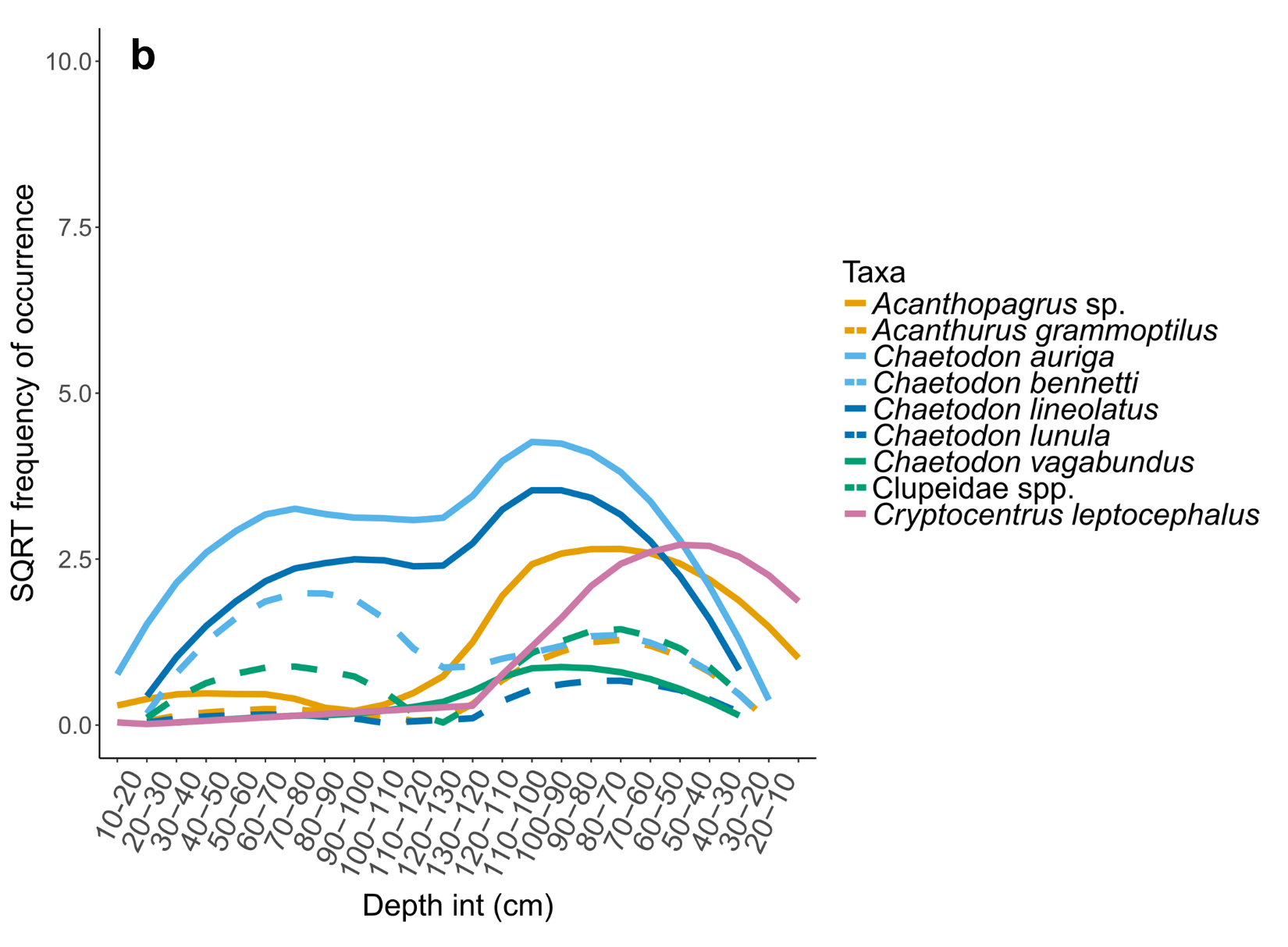

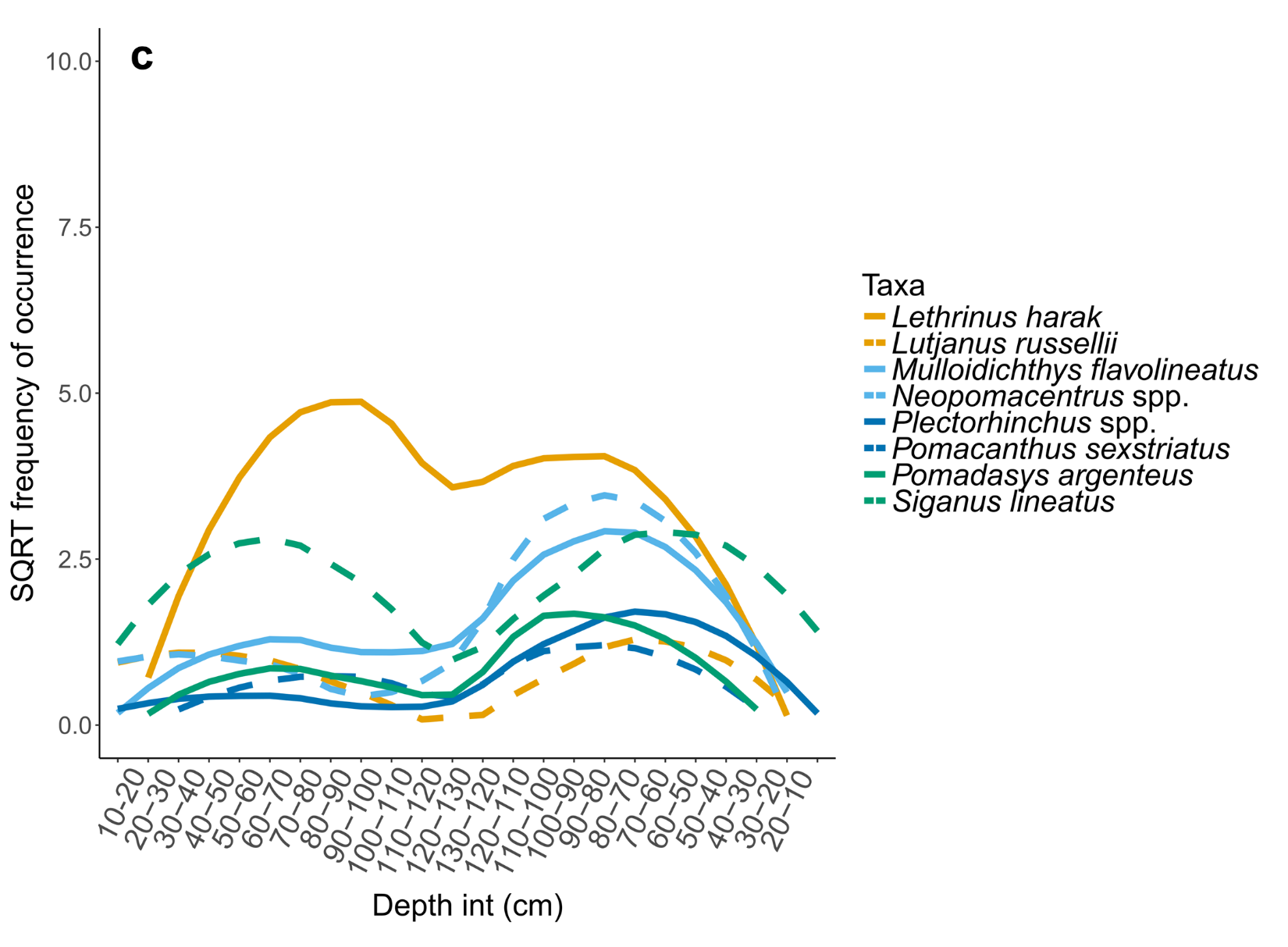

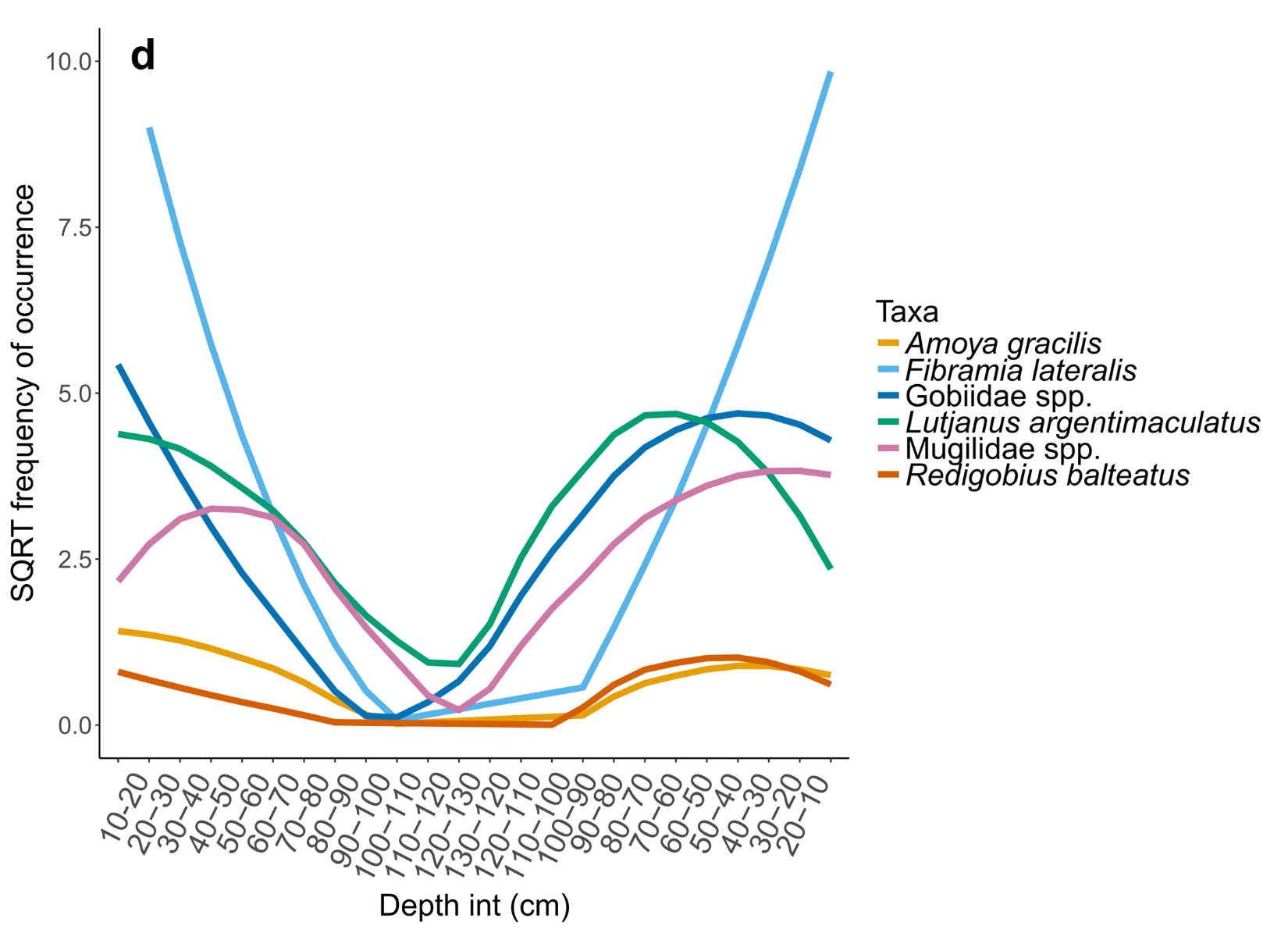

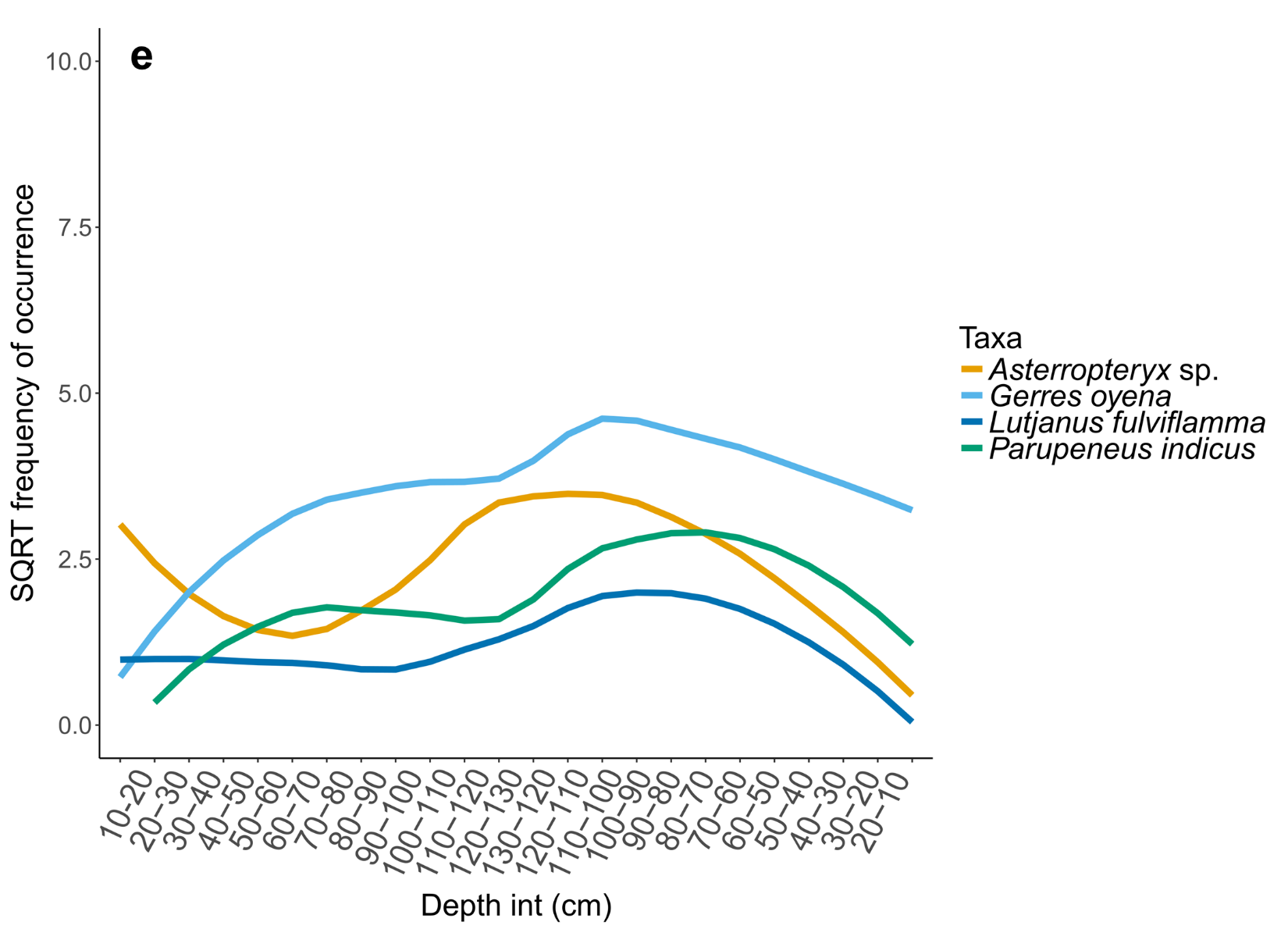

Supplement: S4 Appendix — Species-specific patterns of mangrove utilisation across depth: a) High-depth users; b) Intermediate-depth users; c) Intermediate-depth users (continued) d) Low-depth users; e) Generalist users. (DOCX) [file pone.0207168.s004.docx]
